# Morphology and Developmental Rate of the Blow Fly, *Hemipyrellia ligurriens* (Diptera: Calliphoridae): Forensic Entomology Applications

**DOI:** 10.1155/2012/371243

**Published:** 2012-06-25

**Authors:** Nophawan Bunchu, Chinnapat Thaipakdee, Apichat Vitta, Sangob Sanit, Kom Sukontason, Kabkaew L. Sukontason

**Affiliations:** ^1^Department of Microbiology and Parasitology, Faculty of Medical Science, Naresuan University, Phitsanulok 65000, Thailand; ^2^Centre of Excellence in Medical Biotechnology, Faculty of Medical Science, Naresuan University, Phitsanulok 65000, Thailand; ^3^Department of Parasitology, Faculty of Medicine, Chiang Mai University, Chiang Mai 50200, Thailand

## Abstract

*Hemipyrellia ligurriens* (Diptera: Calliphoridae) is a forensically important blow fly species presented in many countries. In this study, we determined the morphology of all stages and the developmental rate of *H. ligurriens* reared under natural ambient conditions in Phitsanulok province, northern Thailand. Morphological features of all stages based on observing under a light microscope were described and demonstrated in order to use for identification purpose. Moreover, development time in each stage was given. The developmental time of *H. ligurriens* to complete metamorphosis; from egg, larva, pupa to adult, took 270.71 h for 1 cycle of development. The results from this study may be useful not only for application in forensic investigation, but also for study in its biology in the future.

## 1. Introduction

Specimens of blow flies (Diptera: Calliphoridae), especially fly larvae found in corpses and/or at death scenes, can be used as entomological evidence in forensic investigations, that is, estimating the postmortem interval (PMI) and determining toxic substances, antemortem trauma, and whether relocation of remains had occurred [1], as already documented [2–4]. Precise morphological identification of insect specimens is one of very important factors from an applied point of view because they provide relevant evidences for forensic investigations [1]. 

Of blow fly species, *Hemipyrellia ligurriens* (Wiedemann) is a forensically important blow fly species, as reported previously from cases in Thailand [4] and Malaysia [3]. This fly species is distributed widely, covering Korea, Taiwan, Laos, Singapore, Papua New Guinea, Australia, India, China, The Philippines, Sri Lanka, Malaysia, Indonesia, and Thailand [5–7]. Besides its forensic importance, *H*. *ligurriens* can be a nuisance in markets and gardens, and adults also are mechanical vectors of pathogens, due to their attraction to human excreta near human-occupied environments [7]. Previously, morphology of some immature stages (egg, 3rd-instar larvae, and puparium) of *H. ligurriens* has only been studied by observing under a scanning electron microscope and a light microscope [8–13]. For that reason, the available information of *H. ligurriens* is incomplete for identifying all immature evidences which can be found in the death scenes. Moreover, developmental rate of this species which is important data for estimating PMI has not been found in the cited literatures. Moreover, local population-specific developmental data are very important for estimating larval age to determine PMI [14]. To increase the accuracy and precision when applying this information in forensic investigations, the study of all its immature stages and developmental rate is of great interest because the morphology of each one could provide specific features that will become important for a proper identification, and growth data in particular condition could provide essential data for PMI estimation. Therefore, this study aimed to investigate the distinctive characteristics of all its immature stages by observing under the light microscope, to give some important details for identification and to determine its developmental rate, particularly in condition in Phitsanulok province, northern Thailand. 

## 2. Materials and Methods 

### 2.1. Maintenance of *H. ligurriens* in the Laboratory

The colony of *H. ligurriens* used in this study was obtained originally by collecting adult flies with a sweeping net in areas of Sao Hin Village, Muang Phitsanulok district, Phitsanulok province, Thailand (16°44′18N; 100°13′44E). All adults of *H. ligurriens* were collected in the field and identified based on their morphology, using the taxonomic key of Tumrasvin et al. [5], before being reared further in the laboratory. Flies were reared under natural ambient condition in the open-system rearing room, according to the method of Sukontason et al. [8]. Briefly, adults were maintained in a rearing cage (30 × 30 × 30 cm) and fed with 2 kinds of food: (i) a mixture of 10% (w/v) sugar solution and 1.5% (v/v) multivitamin syrup solution (SEVEN SEA, England) and (ii) fresh pork liver. Fresh pork liver was provided as larval food and oviposition site. The presence of eggs on the pork liver was observed daily. When eggs were found, the pork liver having fly eggs were transferred gently into a larva-rearing box by using forceps, and then, 2 or 3 pieces (*≈*50 g/day) of fresh pork liver were added in the box as food for the larvae. Some pieces of fresh pork liver were added daily until the larvae in the box stopped feeding and moving or they became the prepupal stage (late 3rd instar). All pieces of pork liver which remained in the rearing box were removed from the box. Only pupae were kept in the rearing box and sealed tightly until emergence of adult was found. After that, the box was placed and opened in a rearing cage in order to release adults from the box to live in the rearing cage. New fly generation was reared continuously as mentioned above.

### 2.2. Morphology

**Egg**

In this study, egg specimens of *H. ligurriens* were obtained from the laboratory colony to investigate distinct features by using the potassium permanganate staining technique, according to previous description of Sukontason et al. [9]. The main characteristics, such as morphology of median area surrounding the micropyle, and chorionic sculpturing, were observed and photographed under a light microscope (Olympus, Japan), which was connected to a digital camera (Samsung S700, Korea). Furthermore, the widths and lengths of 30 fly eggs were also measured under the calibrated light microscope. Mean and standard deviation of width and length were analyzed by using the Excel program (Microsoft office Enterprise 2007).

**Larva**

This study determined the morphology and developmental rate in all larval stage (1st, 2nd, and 3rd instars). Morphology of all instars was investigated by using the hydroxide clearing method. Briefly, 30 larvae of each instar were obtained from the laboratory colony and sacrificed by placing them into a beaker containing hot water (80°C) for 30 sec to prevent them from shrinking [15]. After that, they were preserved in a small glass bottle containing 70% ethanol. The preserved larvae were dissected individually at two sites to obtain three body portions by using a sharp blade under a stereo microscope (Olympus, Japan), according to the method described by Sukontason et al. [8]. The first cut was positioned across the middle of the second thoracic segment for viewing the internal cephalopharyngeal skeleton and external anterior spiracle. The second cut was positioned across the 11th body segment in order to observe the characteristics of the posterior spiracle. As a clearing method, each pair of anterior and posterior parts was left in a glass plate containing 10% (w/v) potassium hydroxide solution for 1 day (for 1st instar) or 2 days (for 2nd and 3rd instars). Subsequently, the specimens were washed twice with distilled water and neutralized by placing them on a glass plate containing a mixture of 35% ethanol and 1% glacial acetic acid for 30 min. After that, the specimens were dehydrated serially in 50%, 70%, 80%, 95%, and absolute ethanol (RCI LABSCAN, Thailand) for 30 min per alcohol concentration. Dehydrated specimens were transferred onto a glass plate containing xylene (PROLAB, France) and left for 1 min before mounting on a glass slide with 2-3 drops of mounting medium (Permount). A cover slip was placed over each specimen. The permanent slides were left in room temperature for 2 days before observing under a light microscope. The cephalopharyngeal skeleton and posterior spiracle of each instar were photographed under the light microscope, which was connected to the digital camera. In addition, the body length and width of all instars were examined. Thirty of the first instar larvae were measured by using a calibrated microscope, while the second and third instars (*n* = 30 larvae/ each instar) were measured with vernier calipers under a dissecting microscope. Mean and standard deviation of their body width and length were analyzed by using the Excel program.

**Pupa**

In this stage, morphology of the anterior and posterior parts and coloration change were studied. The anterior and posterior parts of puparia were determined by using the potassium hydroxide clearing technique, previously described by Sukontason et al. [10]. The anterior parts of puparia were the remnants of the contracted head to the fourth segment of puparia after the flies had emerged. They were recruited from the rearing box that flies had already emerged. For each posterior part, the caudal segment of puparium was cut with a sharp blade under the stereo microscope and then transferred by using forceps into the same glass plate as that used for the anterior part. After that, they were soaked in 1% (v/v) dish-washing detergent to remove surface artifacts and/or pork liver tissues. As a clearing method, the anterior and posterior parts were left in a test tube containing 10% (w/v) potassium hydroxide solution, and the test tube was transferred into a water bath set at a temperature of 80°C for 1 h. Then, the specimen process was performed following that described for the larva stage. The important features of anterior and posterior parts were observed and photographed under the light microscope, connected to the digital camera. Number of papillae in each posterior spiracle of 30 anterior parts was counted and calculated for its range. In addition, thirty puparia were measured for their width and length by using vernier calipers. Mean and standard deviation of their width and length were analyzed by using the Excel program. For observation of coloration change, only one puparium which represented the color of the puparia in the rearing box was selected and photographed at 0, 3, 6, 9, 12, 15, 18, 21, and 24 h by using the digital camera. Before taking photographs, puparium was washed in distilled water to remove surface artifacts.

**Adult**

The young adults, 5–7days old, were obtained from the rearing caged by using a test tube. They were sacrificed by placing them in a refrigerator (−4°C) for 2 h. After that, they were gently pinned with good anatomical arrangement. The important characteristics for identification were examined and photographed under the stereo microscope, connected to the digital camera. In addition, thirty adults were measured for body width and length by using vernier calipers. In this study, body width means the width of the 2nd thoracic segment, and body length is the entire length of the body, ranging from the middle compound eye to the last segment of abdomen. Mean and standard deviation of their body width and length were analyzed by using the Excel program.

### 2.3. Developmental Rate Assessment

For assessment of the developmental rate, the experiments were performed in the open-system rearing room under the natural ambient temperature of Phitsanulok province, northern Thailand. Temperature and relative humidity were recorded daily by using a thermometer and hygrometer (Thermo-Hygro TM870, China). The ranges (Mean ± SD) of recorded temperature and relative humidity used to determine the development rate of this fly were 26.7 ± 0.61°C and 74 ± 3%, respectively. For each experiment, the developmental rate began at finding of newly hatched larvae (or the 1st instar); the prepupal stage signified the endpoint. The newly hatched larvae were recognized as 0 h old larvae. Developmental rate assessment in this study was studied by separating the newly hatched larvae from the same adult fly colony into 3 groups. Each group consisted of 150–200 newly hatched larvae. They were transferred gently from the rearing box to the new one by using a wet paint brush (number 4). Fresh pork liver (*≈*50 g) was provided daily as a food source for the larvae in each rearing box. The five largest larvae were removed from the rearing box every 3 h. They were sacrificed by placing in hot water (*≈*80°C) for 30 seconds to prevent larval shrinkage [15] and were preserved in a small glass bottle containing 70% ethanol. All preserved larvae were measured body lengths by using a calibrated microscope or vernier calipers under a dissecting microscope, depending on their sizes. The relation of larval body lengths and developmental time was analyzed using the Excel program. In addition, life spans in other stages were recorded and analyzed by using the Excel program. 

## 3. Results

### 3.1. Morphology

**Egg**

**Figure 1:**
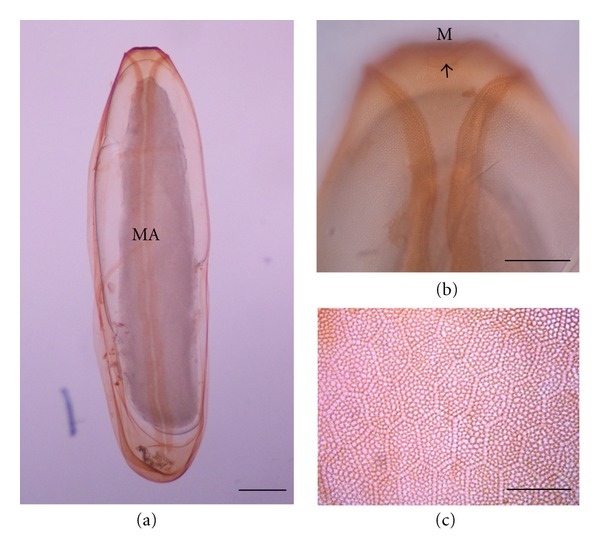
Egg of *Hemipyrellia ligurriens* stained with 1% potassium permanganate. (a) Whole egg; MA: median area. (b) Anterior end of egg showing bifurcated plastron and micropyle; M: micropyle. (c) External chorionic sculpture showing hexagonal pattern, with its reticular boundary slightly elevating like a net. Bars = 100 *μ*m for all figures.

The egg of *H. ligurriens* was elongated and tapered at both anterior and posterior ends (Figure 1(a)). It measured 1.44 ± 0.11 mm in length and 0.47 ± 0.04 mm in width (Table 1). It took time in this stage for 10.3 ± 0.30 h before moulting to be larval stage (Table 1). The unstained egg was creamy white; while those after staining with 1% potassium permanganate were light brown in color. The median area was located dorsally, positioning a Y-shape that extended from the anterior end to almost the posterior end (Figure 1(b)). The hatching line was upright, thus displaying dark thickening along the median area (Figure 1(a)). The chorionic sculpture appeared as a hexagonal pattern, with its reticular boundary slightly elevating like a net (Figure 1(c)).

**Larva**

**Figure 2:**
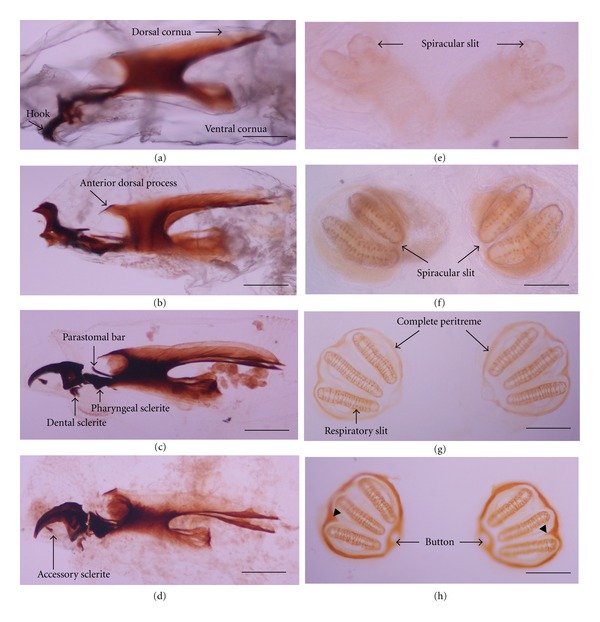
Cephalopharyngeal skeletons and posterior spiracles of *Hemipyrellia ligurriens* larvae. Cephalopharyngeal skeletons of (a) 1st instar, (b) 2nd instar, (c) early 3rd instar,. and (d) late 3rd instar. Posterior spiracles of (e) 1st instar, (f) 2nd instar, (g) early 3rd instar, and (h) late 3rd instar. Bars = 100 *μ*m for all figures.

Under observation with the stereo microscope, all larval instars of *H. ligurriens* displayed typical muscoid-shaped vermiform larva that was pointed in anterior and blunt in posterior. The caudal segment had a single pair of posterior spiracles. The first instar was relatively small, measuring 2.62 ± 0.70 mm in length and 0.75 ± 0.35 mm in width, with the developmental time at this stage for 12 ± 0.1 h (Table 1). The cephalopharyngeal skeleton was not well developed (Figure 2(a)), while the posterior spiracle had 2 spiracular slits merging ventrally (Figure 2(e)). The second instar was 6.24 ± 1.67 mm in length and 1.37 ± 0.19 mm in width, with 12 ± 3 h duration at this stage (Table 1). The cephalopharyngeal skeleton was almost complete (Figure 2(b)), while the posterior spiracle had 2 separated spiracular slits with weakly pigmented incomplete peritreme (Figure 2(f)). The size of the 3rd instar was the largest, measuring 12.18 ± 1.31 mm in length and 2.32 ± 0.19 mm in width. The developmental time was also the longest at this stage, being 84 ± 3 h (Table 1). The cephalopharyngeal skeletons of the early and the late 3rd instar were similar (Figures 2(c) and 2(d), resp.). The posterior spiracles of the early (Figure 2(g)) and late 3rd instar (Figure 2(h)) were similar, having 3 separated spiracular slits and complete peritreme with an interslit projection, except for the latter showing a highly pigmented peritreme (Figure 2(h)). The distinctive features of all three instars were summarized in Table 2.

**Pupa**

**Figure 3:**
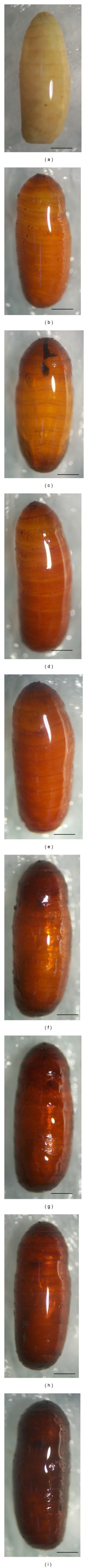
Color changes in puparia of *Hemipyrellia ligurriens* up to 24 h after pupariation. Puparium (a)–(i) at 0, 3, 6, 9, 12, 15, 18, 21, and 24 h, respectively. Bars = 100 *μ*m for all figures.

The puparium of *H. ligurriens* was of typical coarctate form (Figure 3), which measured 6.82 ± 0.27 mm in length and 2.76 ± 0.11 mm in width (Table 1). Observed color changes revealed that the early puparium was creamy white in color (0 h), with the posterior end still truncated (Figure 3(a)). However, the color of the puparium changed gradually to light yellow brown within 3 h after pupariation (Figure 3(b)) and then to brown after around 15 h (Figure 3(f)). Color of puparium changed to dark brown after about 18 h of observation (Figure 3(g)). At 24 h, the puparium showed the darkest brown in color (Figure 3(i)). The duration for the entire pupal stage was 152.5 ± 30.7 h (Table 1).

**Figure 4:**
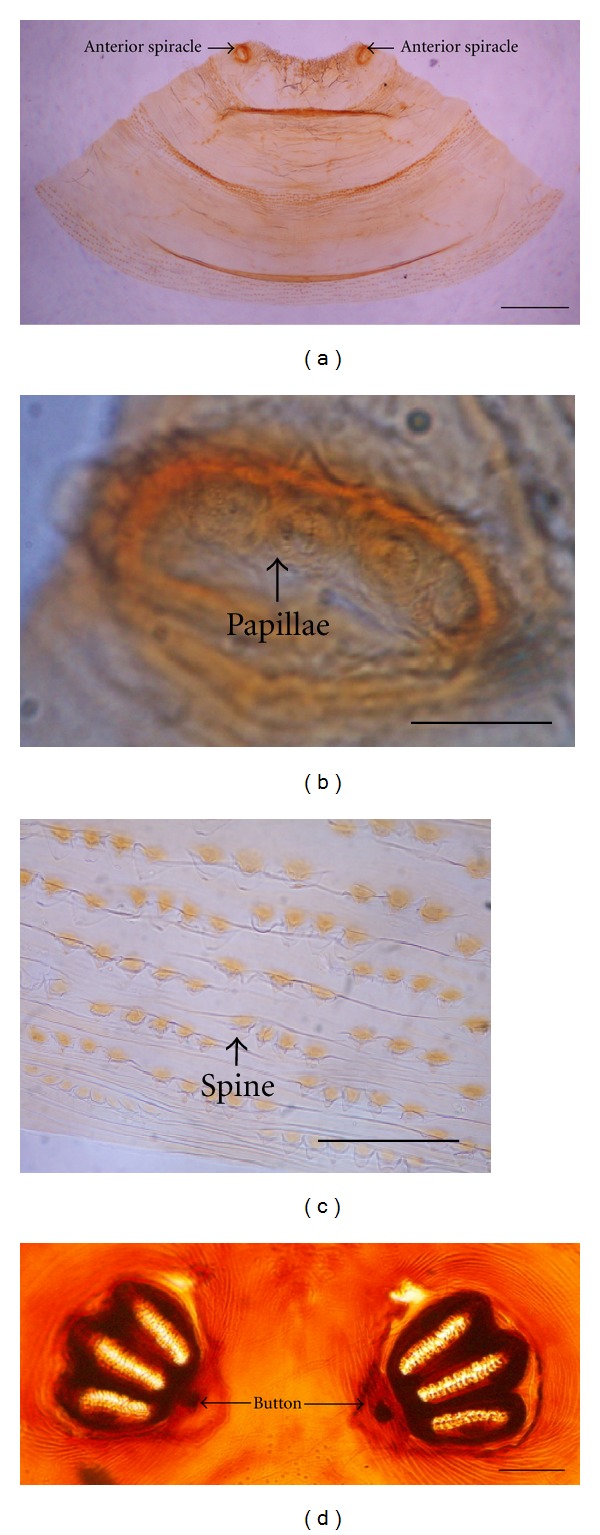
Puparium of *Hemipyrellia ligurriens* after treating with 10% KOH. (a) Anterior plate with trapezoid shape. (b) Anterior spiracle with 6 papillae. (c) Spine pattern at the 3rd segment showing single point tip. (d) Posterior spiracles. Bars = 100 *μ*m for all figures.

Observation of the anterior and posterior parts of puparia appeared as a pale yellow-brown color after being treated with 10% potassium hydroxide. The anterior plate, pressed with a cover slip, was trapezoid-shaped with 2 anterior spiracles located at both top ends (Figure 4(a)). Each anterior spiracle consisted of 5 to 7 papillae (Figure 4(b)). The spine observed in the third segment displayed rows of single-pointed tips (Figure 4(c)). Each posterior spiracle appeared three highly pigmented dark brown spiracular slits, with highly pigmented button and weakly pigmented peritreme (Figure 4(d)).

**Adult**

**Figure 5:**
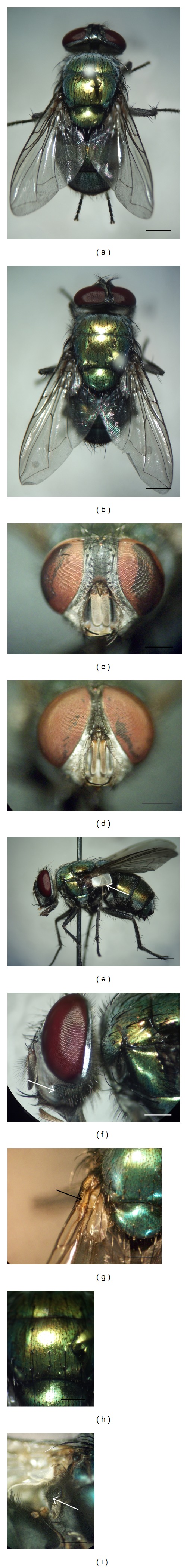
Adults and important characteristics for identification of *Hemipyrellialigurrien*. (a) Dorsal view of the female. (b) Dorsal view of the male. (c) Frons of the female showing dichoptic eyes. (d) Frons of the male showing subholoptic eyes. (e) Lateral view of the adult showing whitish squamae (arrow). (f) Higher magnification of the head showing black hairs on the gena (arrow). (g) Wing showing the stem vein without setulae (arrow). (h) Dorsal thorax. (i) Pilose hair on the supraconvexity (arrow). Bars = 200 *μ*m for all figures.

The adult* H. ligurriens* was 12.23 ± 0.61 mm in length, 2.85 ± 0.25 mm in width (Table 1), and metallic copper green in appearance, with grayish white pollinose on the anterior part of the thorax (Figures 5(a) and 5(b)). Head of the female was dichoptic, but that of male was subholoptic (Figures 5(c) and 5(d)). Grayish facial pollinosity was found in both sexes (Figures 5(c) and 5(d)), and the entire third antennal segment was dark brown or orange ventrally (Figures 5(c) and 5(d)). Squama was whitish (Figure 5(e)). Gena was covered with black hairs (Figure 5(f)). Stem vein was without setulae (Figure 5(g)). Two postsutural acrostichal setae were found on the thorax (Figure 5(h)). Supraconvexity was found pilose hair (Figure 5(i)). All above characteristics were important for identification of this fly adult.

### 3.2. Developmental Rate Assessment

**Figure 6:**
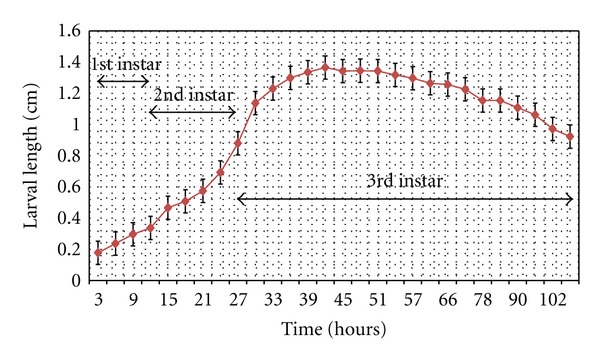
Relation between developmental time and the larval length of *Hemipyrellia ligurriens* under natural ambient conditions (26.7 ± 0.61°C; 74 ± 3% RH) of Phitsanulok province, northern Thailand.

Assessments of the developmental rate of *H.ligurriens* were determined during the investigation period, based on natural ambient condition of Phitsanulok province. The growth curve of larval stage showed sigmoid form (Figure 6). Data on the developmental rate of rearing revealed that developmental time from newly hatched larvae to beginning of pupariation grows rapidly in a total period of 108 h. Larvae reached their maximum median length at *≈*42 h, and pupariation occurred at 108h. The life spans of all stages were also summarized in Table 1.

## 4. Discussion

Data related to important insect characteristics for fast and reliable identification and development in the laboratory are crucial prerequisites for appropriate application in forensic investigations. *H. ligurriens* is a forensically important blow fly species that has been recorded in forensic cases in Malaysia and Thailand [3, 4]. This study focused on the important characteristics for identifying all stages of *H. ligurriens,* based on observation under the light microscope and developmental time of each stage in natural ambient condition with average temperatures and relative humidity, which were 26.7 ± 0.61°C and 74 ± 3%, respectively. Although morphology at some stages of *H. ligurriens* has been studied previously using the scanning electron microscope and the light microscope [8–13], this study provided more detailed information on distinctive features and new clues for identification by using simple techniques in order to increase accuracy and precision of forensic application in the future. Moreover, this study provided developmental data of *H. ligurriens*, which can be used in estimating the PMI of corpses on which this fly species has colonized.

Chorionic sculpture as well as width and length of median area were reported previously as an important characteristic in identifying the fly egg [9, 13]. Morphology of the *H. ligurriens* egg, observed in this study by staining with 1% potassium permanganate and observing under the light microscope, was similar to that in previous work, which investigated by using the scanning electron microscope [11]. Therefore, results from this study confirmed the effectiveness of staining method, initiated by Sukontason et al. [9], for identification purpose of fly eggs. The stained eggs could be observed clearly under the light microscope. When comparing data with previous studies, the size of the *H. ligurriens* egg in this study was larger than that of other blow flies, such as *Lucilia cuprina*, *Ceylonomyia *(= *Chrysomya*) *nigripes*, and *Aldrichina grahami*; however, its size was not different from the sizes of* Chrysomya megacephala* and* Achoetandrus* (=  *Chrysomya*) *rufifacies* [9]. Nevertheless, size of fly egg cannot be used as the primary characteristics for identification, according to information from Erzinclioglu [16], who reported that the size of fly egg depended on the dietary levels. Therefore, identification of fly egg should use characteristics of the width of plastron, morphology of plastron area surrounding the micropyle as the main criteria and use size as a supplemental feature for egg identification [9]. 

Based on our references, this study was the first study, showing morphology of cephalopharyngeal skeleton and posterior spiracle in all instar larvae by observing under the light microscope. Cephalopharyngeal skeletons and posterior spiracles of larvae were seen clearly as being different in each instar. The results of this study agreed with the previous reports [11–13]. In addition, this study showed degree of pigmentation of peritreme in each instar. It may be a new clue for differentiating age of larvae, especially in between early and late instar. With each developmental instar definitely being different as well as the degree of pigmentation, the data from this study may be useful in identifying the larval stages and age of larvae of this blow fly in detail as mentioned above. 

Study on morphology of pupal stage provided the results which were similar to the previous reports [10]. The results from observation in coloration changes by time of *H. ligurriens* puparia were firstly reported in this study and may be another new supportive evidence to determine at least the approximate age of puparia for increasing accuracy of PMI value. 

This study demonstrated some photographs of distinctive features for using in identification of adult *H. ligurriens*. These photographs of peculiar characteristics from this study may be useful for people who are not familiar with the terminology in the taxonomic keys. Current taxonomic key for identification of blow fly species in Thailand was given by Kurahashi and Bunchu [17]. By the naked eyes of a nonentomologist, adult *H. ligurriens* seems similar to *A. rufifacies* in appearance. Therefore, the chance of misidentification may occur in both species, especially in Thailand because *A. rufifacies* was the second most predominant blow fly species of Thailand, and coincidence of both species in some provinces in this country was found [18]. 

This study was the first report that provided developmental time of *H. ligurriens* in all stages. The results showed that the developmental time of *H. ligurriens* under natural conditions in this study (an average temperature of 26.7 ± 0.61°C and relative humidity of 74 ± 3%) was faster than that of *C. megacephala *and  *A*. *rufifacies,* which were studied at an average temperature of 27.4°C [19]. Furthermore, the developmental time of each stage of *H. ligurriens* was different from that of *C. megacephala *and  *A*. *rufifacies*. The developmental rate of each species was specified, although they grew in the same or related conditions [18]. Furthermore, variation of developmental times within a blow fly species was found in geographically different populations [14]. The local population-specific developmental data are needed for estimating larval age to determine PMI. Therefore, the data from this study are very important for further application, particularly in Phitsanulok province.

Data, obtained from both the morphological characteristics and developmental time, fulfill the previous information and provide new supportive evidences for identification of this blow fly species and application in forensic investigation, particularly when *H. ligurriens* is present in human cadavers. Moreover, they may be useful as baseline data in its biology study in the future. 

## Figures and Tables

**Table 1:** Size and life span (Mean ± SD) of each developmental stage of *Hemipyrellia ligurriens* under natural ambient conditions (26.7 ± 0.61^°^C, 74 ± 3% RH) of Phitsanulok province, northern Thailand.

Stage	Length (Mean ± SD)	Width (Mean ± SD)	Duration (Mean ± SD)
Egg	1.44 ± 0.11 mm	0.47 ± 0.04 mm	10.3 ± 0.30 hrs
1st instar	2.62 ± 0.70 mm	0.75 ± 0.35 mm	12.0 ± 0.10 hrs
2nd instar	6.24 ± 1.67 mm	1.37 ± 0.19 mm	12.0 ± 3.00 hrs
3rd instar	12.18 ± 1.31 mm	2.32 ± 0.19 mm	84.0 ± 3.00 hrs
Pupa	6.82 ± 0.27 mm	2.76 ± 0.11 mm	152.5 ± 30.70 hrs
Adult	12.23 ± 0.61 mm	2.85 ± 0.25 mm	270.7 ± 30.90 hrs

**Table 2:** Comparison of distinctive features of the 1st, 2nd, and 3rd instar larvae of *Hemipyrellia ligurriens. *

Character	1st instar	2nd instar	3rd instar
Cephalopharyngeal skeletal:			
accessory sclerite of cephalopharyngeal skeletal	Absent	Absent	Present
Posterior spiracle:			
button of posterior spiracle	Absent	Absent	Present
peritreme of posterior spiracle	Very weakly pigmented; incomplete peritreme	Weakly pigmented; incomplete peritreme without an interslit projection	Highly pigmented; complete peritreme with an interslit projection
Number of spiracular slit	2 slits merging ventrally	2 separated slits	3 separated slits
